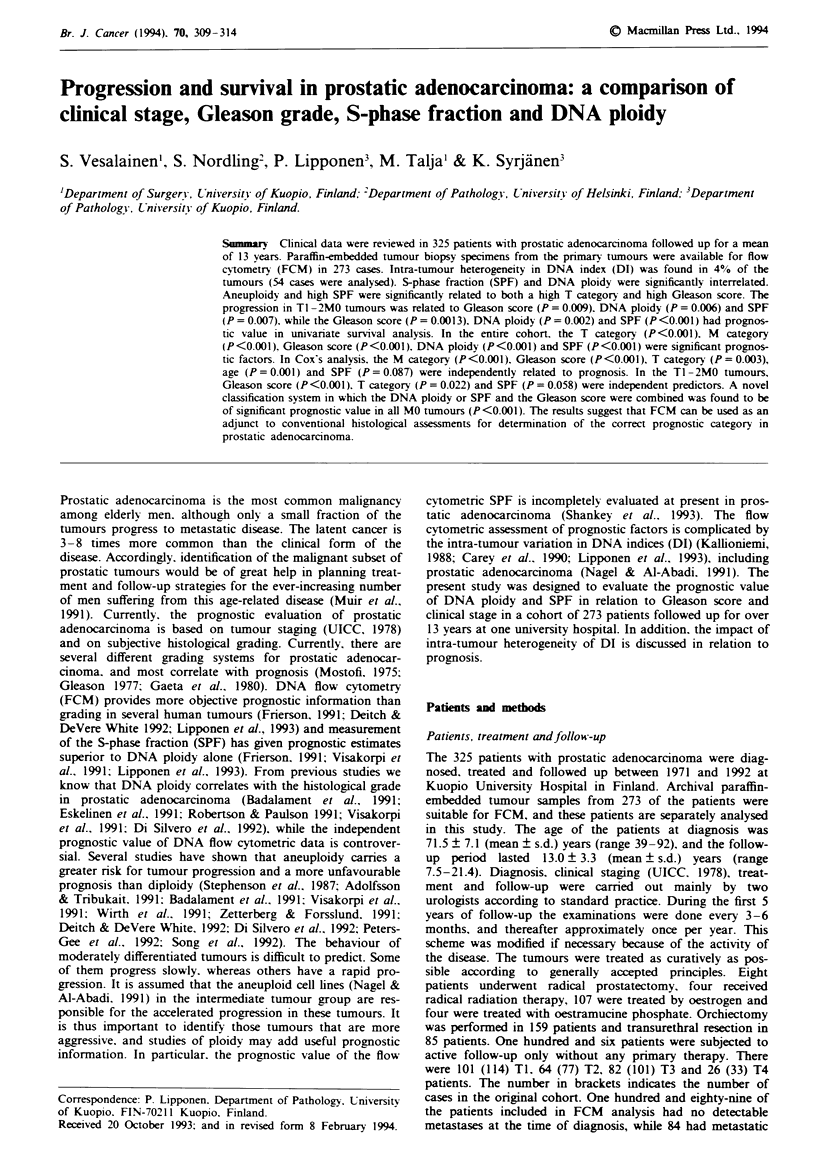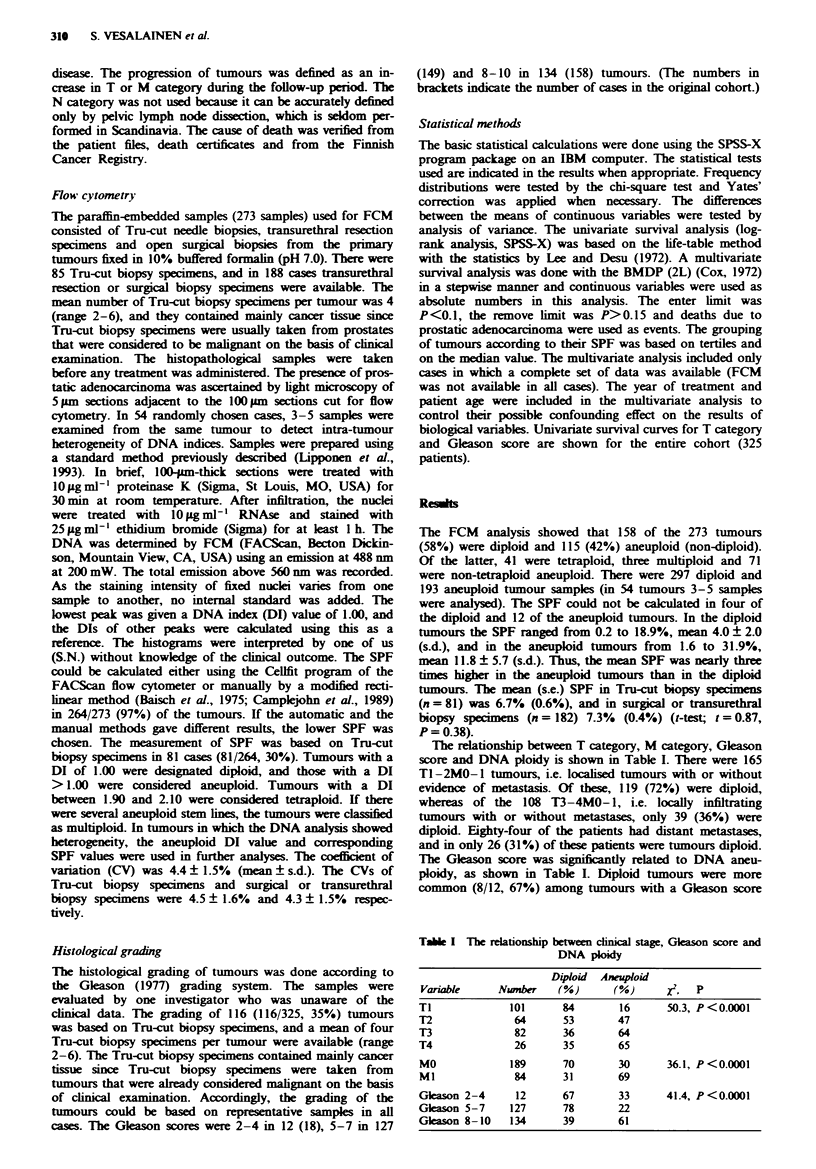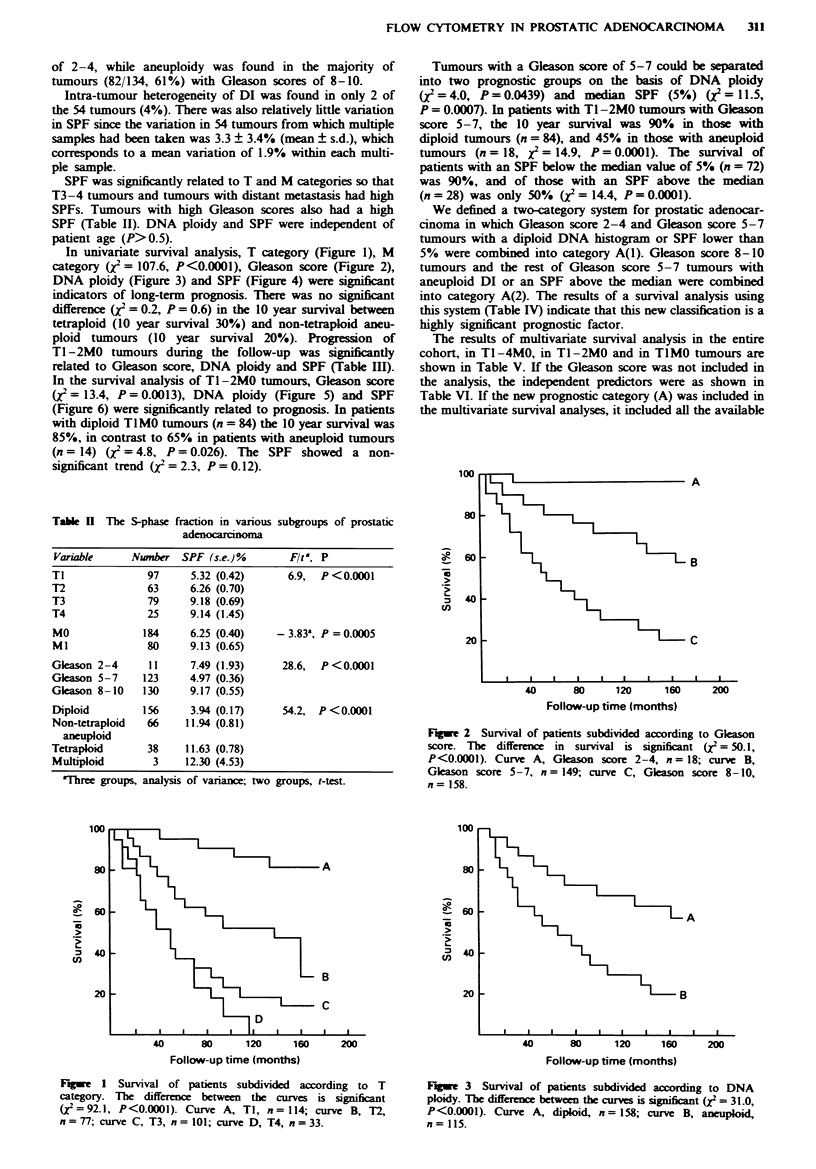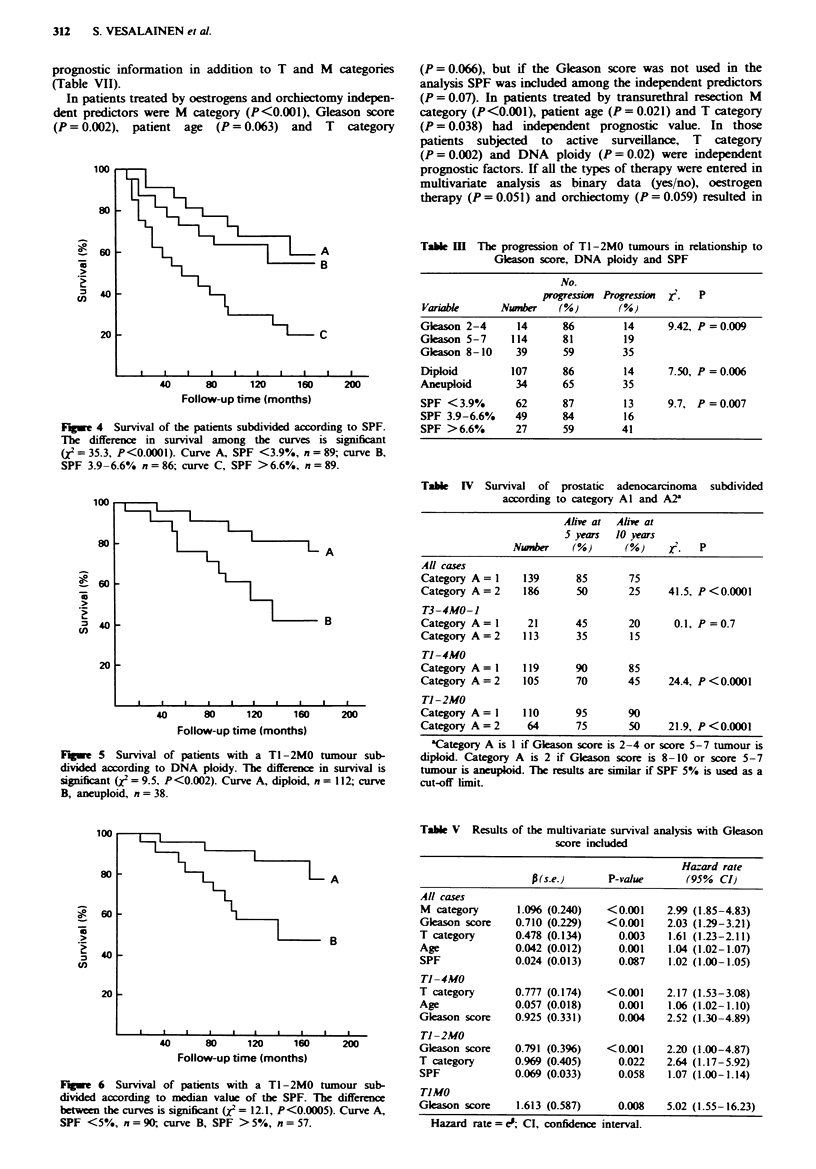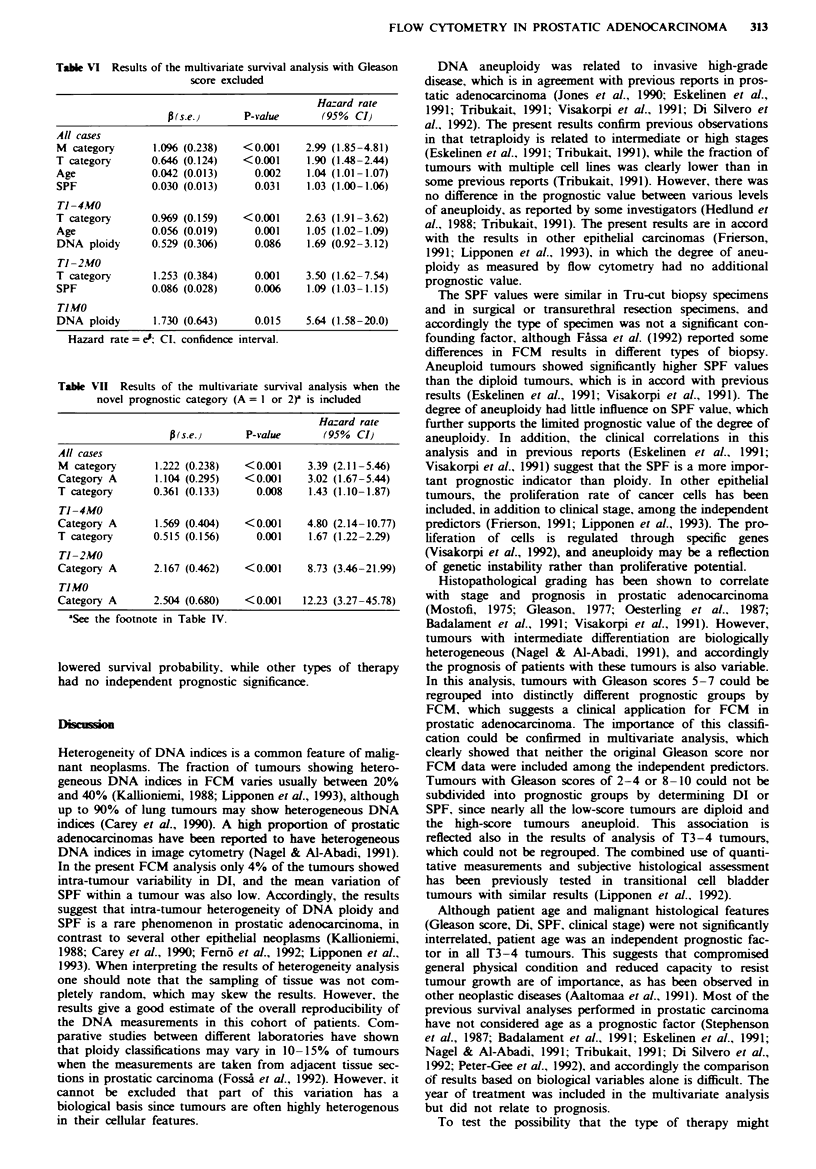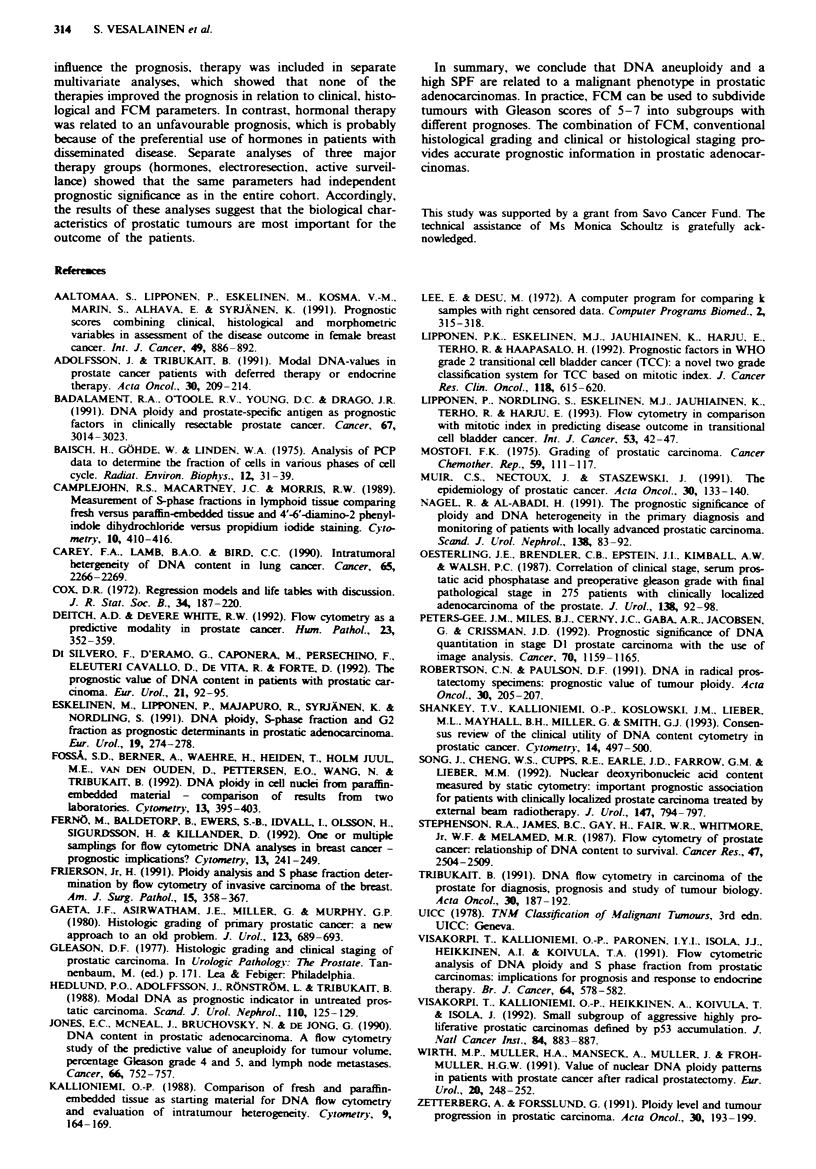# Progression and survival in prostatic adenocarcinoma: a comparison of clinical stage, Gleason grade, S-phase fraction and DNA ploidy.

**DOI:** 10.1038/bjc.1994.298

**Published:** 1994-08

**Authors:** S. Vesalainen, S. Nordling, P. Lipponen, M. Talja, K. Syrjänen

**Affiliations:** Department of Surgery, University of Kuopio, Finland.

## Abstract

Clinical data were reviewed in 325 patients with prostatic adenocarcinoma followed up for a mean of 13 years. Paraffin-embedded tumour biopsy specimens from the primary tumours were available for flow cytometry (FCM) in 273 cases. Intra-tumour heterogeneity in DNA index (DI) was found in 4% of the tumours (54 cases were analysed). S-phase fraction (SPF) and DNA ploidy were significantly interrelated. Aneuploidy and high SPF were significantly related to both a high T category and high Gleason score. The progression in T1-2M0 tumours was related to Gleason score (P = 0.009), DNA ploidy (P = 0.006) and SPF (P = 0.007), while the Gleason score (P = 0.0013), DNA ploidy (P = 0.002) and SPF (P < 0.001) had prognostic value in univariate survival analysis. In the entire cohort, the T category (P < 0.001), M category (P < 0.001), Gleason score (P < 0.001), DNA ploidy (P < 0.001) and SPF (P < 0.001) were significant prognostic factors. In Cox's analysis, the M category (P < 0.001), Gleason score (P < 0.001), T category (P = 0.003), age (P = 0.001) and SPF (P = 0.087) were independently related to prognosis. In the T1-2M0 tumours, Gleason score (P < 0.001), T category (P = 0.022) and SPF (P = 0.058) were independent predictors. A novel classification system in which the DNA ploidy or SPF and the Gleason score were combined was found to be of significant prognostic value in all M0 tumours (P < 0.001). The results suggest that FCM can be used as an adjunct to conventional histological assessments for determination of the correct prognostic category in prostatic adenocarcinoma.


Br. J. Cancer (1994), 70, 309-314 © Macmillan Press Ltd., 1994

### Progression and survival in prostatic adenocarcinoma: a comparison of clinical stage, Gleason grade, S-phase fraction and DNA ploidy

S. Vesalainen[1], S. Nordling[2], P. Lipponen[3], M. Talja[1] & K. Syrjänen[3]

[1]Department of Surgery, University of Kuopio, Finland; [2]Department of Pathology, University of Helsinki, Finland; [3]Department of Pathology, University of Kuopio, Finland.

**Summary** Clinical data were reviewed in 325 patients with prostatic adenocarcinoma followed up for a mean of 13 years. Paraffin-embedded tumour biopsy specimens from the primary tumours were available for flow cytometry (FCM) in 273 cases. Intra-tumour heterogeneity in DNA index (DI) was found in 4% of the tumours (54 cases were analysed). S-phase fraction (SPF) and DNA ploidy were significantly interrelated. Aneuploidy and high SPF were significantly related to both a high T category and high Gleason score. The progression in T1-2M0 tumours was related to Gleason score ($P = 0.009$), DNA ploidy ($P = 0.006$) and SPF ($P = 0.007$), while the Gleason score ($P = 0.0013$), DNA ploidy ($P = 0.002$) and SPF ($P<0.001$) had prognostic value in univariate survival analysis. In the entire cohort, the T category ($P<0.001$), M category ($P<0.001$), Gleason score ($P<0.001$), DNA ploidy ($P<0.001$) and SPF ($P<0.001$) were significant prognostic factors. In Cox's analysis, the M category ($P<0.001$), Gleason score ($P<0.001$), T category ($P = 0.003$), age ($P = 0.001$) and SPF ($P = 0.087$) were independently related to prognosis. In the T1-2M0 tumours, Gleason score ($P<0.001$), T category ($P = 0.022$) and SPF ($P = 0.058$) were independent predictors. A novel classification system in which the DNA ploidy or SPF and the Gleason score were combined was found to be of significant prognostic value in all M0 tumours ($P<0.001$). The results suggest that FCM can be used as an adjunct to conventional histological assessments for determination of the correct prognostic category in prostatic adenocarcinoma.

Prostatic adenocarcinoma is the most common malignancy among elderly men, although only a small fraction of the tumours progress to metastatic disease. The latent cancer is 3-8 times more common than the clinical form of the disease. Accordingly, identification of the malignant subset of prostatic tumours would be of great help in planning treatment and follow-up strategies for the ever-increasing number of men suffering from this age-related disease (Muir *et al.*, 1991). Currently, the prognostic evaluation of prostatic adenocarcinoma is based on tumour staging (UICC, 1978) and on subjective histological grading. Currently, there are several different grading systems for prostatic adenocarcinoma, and most correlate with prognosis (Mostofi, 1975; Gleason 1977; Gaeta *et al.*, 1980). DNA flow cytometry (FCM) provides more objective prognostic information than grading in several human tumours (Frierson, 1991; Deitch & DeVere White 1992; Lipponen *et al.*, 1993) and measurement of the S-phase fraction (SPF) has given prognostic estimates superior to DNA ploidy alone (Frierson, 1991; Visakorpi *et al.*, 1991; Lipponen *et al.*, 1993). From previous studies we know that DNA ploidy correlates with the histological grade in prostatic adenocarcinoma (Badalament *et al.*, 1991; Eskelinen *et al.*, 1991; Robertson & Paulson 1991; Visakorpi *et al.*, 1991; Di Silvero *et al.*, 1992), while the independent prognostic value of DNA flow cytometric data is controversial. Several studies have shown that aneuploidy carries a greater risk for tumour progression and a more unfavourable prognosis than diploidy (Stephenson *et al.*, 1987; Adolfsson & Tribukait, 1991; Badalament *et al.*, 1991; Visakorpi *et al.*, 1991; Wirth *et al.*, 1991; Zetterberg & Forsslund, 1991; Deitch & DeVere White, 1992; Di Silvero *et al.*, 1992; Peters-Gee *et al.*, 1992; Song *et al.*, 1992). The behaviour of moderately differentiated tumours is difficult to predict. Some of them progress slowly, whereas others have a rapid progression. It is assumed that the aneuploid cell lines (Nagel & Al-Abadi, 1991) in the intermediate tumour group are responsible for the accelerated progression in these tumours. It is thus important to identify those tumours that are more aggressive, and studies of ploidy may add useful prognostic information. In particular, the prognostic value of the flow cytometric SPF is incompletely evaluated at present in prostatic adenocarcinoma (Shankey *et al.*, 1993). The flow cytometric assessment of prognostic factors is complicated by the intra-tumour variation in DNA indices (DI) (Kallioniemi, 1988; Carey *et al.*, 1990; Lipponen *et al.*, 1993), including prostatic adenocarcinoma (Nagel & Al-Abadi, 1991). The present study was designed to evaluate the prognostic value of DNA ploidy and SPF in relation to Gleason score and clinical stage in a cohort of 273 patients followed up for over 13 years at one university hospital. In addition, the impact of intra-tumour heterogeneity of DI is discussed in relation to prognosis.

Correspondence: P. Lipponen, Department of Pathology, University of Kuopio, FIN-70211 Kuopio, Finland.
Received 20 October 1993; and in revised form 8 February 1994.

### Patients and methods

### *Patients, treatment and follow-up*

The 325 patients with prostatic adenocarcinoma were diagnosed, treated and followed up between 1971 and 1992 at Kuopio University Hospital in Finland. Archival paraffin-embedded tumour samples from 273 of the patients were suitable for FCM, and these patients are separately analysed in this study. The age of the patients at diagnosis was 71.5 ± 7.1 (mean ± s.d.) years (range 39-92), and the follow-up period lasted 13.0 ± 3.3 (mean ± s.d.) years (range 7.5-21.4). Diagnosis, clinical staging (UICC, 1978), treatment and follow-up were carried out mainly by two urologists according to standard practice. During the first 5 years of follow-up the examinations were done every 3-6 months, and thereafter approximately once per year. This scheme was modified if necessary because of the activity of the disease. The tumours were treated as curatively as possible according to generally accepted principles. Eight patients underwent radical prostatectomy, four received radical radiation therapy, 107 were treated by oestrogen and four were treated with oestramucine phosphate. Orchiectomy was performed in 159 patients and transurethral resection in 85 patients. One hundred and six patients were subjected to active follow-up only without any primary therapy. There were 101 (114) T1, 64 (77) T2, 82 (101) T3 and 26 (33) T4 patients. The number in brackets indicates the number of cases in the original cohort. One hundred and eighty-nine of the patients included in FCM analysis had no detectable metastases at the time of diagnosis, while 84 had metastatic disease. The progression of tumours was defined as an increase in T or M category during the follow-up period. The N category was not used because it can be accurately defined only by pelvic lymph node dissection, which is seldom performed in Scandinavia. The cause of death was verified from the patient files, death certificates and from the Finnish Cancer Registry.

### *Flow cytometry*

The paraffin-embedded samples (273 samples) used for FCM consisted of Tru-cut needle biopsies, transurethral resection specimens and open surgical biopsies from the primary tumours fixed in 10% buffered formalin (pH 7.0). There were 85 Tru-cut biopsy specimens, and in 188 cases transurethral resection or surgical biopsy specimens were available. The mean number of Tru-cut biopsy specimens per tumour was 4 (range 2–6), and they contained mainly cancer tissue since Tru-cut biopsy specimens were usually taken from prostates that were considered to be malignant on the basis of clinical examination. The histopathological samples were taken before any treatment was administered. The presence of prostatic adenocarcinoma was ascertained by light microscopy of 5 $\mu m$ sections adjacent to the 100 $\mu m$ sections cut for flow cytometry. In 54 randomly chosen cases, 3–5 samples were examined from the same tumour to detect intra-tumour heterogeneity of DNA indices. Samples were prepared using a standard method previously described (Lipponen *et al.*, 1993). In brief, 100-$\mu m$-thick sections were treated with 10 $\mu g\ ml^{-1}$ proteinase K (Sigma, St Louis, MO, USA) for 30 min at room temperature. After infiltration, the nuclei were treated with 10 $\mu g\ ml^{-1}$ RNAse and stained with 25 $\mu g\ ml^{-1}$ ethidium bromide (Sigma) for at least 1 h. The DNA was determined by FCM (FACScan, Becton Dickinson, Mountain View, CA, USA) using an emission at 488 nm at 200 mW. The total emission above 560 nm was recorded. As the staining intensity of fixed nuclei varies from one sample to another, no internal standard was added. The lowest peak was given a DNA index (DI) value of 1.00, and the DIs of other peaks were calculated using this as a reference. The histograms were interpreted by one of us (S.N.) without knowledge of the clinical outcome. The SPF could be calculated either using the Cellfit program of the FACScan flow cytometer or manually by a modified rectilinear method (Baisch *et al.*, 1975; Camplejohn *et al.*, 1989) in 264/273 (97%) of the tumours. If the automatic and the manual methods gave different results, the lower SPF was chosen. The measurement of SPF was based on Tru-cut biopsy specimens in 81 cases (81/264, 30%). Tumours with a DI of 1.00 were designated diploid, and those with a DI >1.00 were considered aneuploid. Tumours with a DI between 1.90 and 2.10 were considered tetraploid. If there were several aneuploid stem lines, the tumours were classified as multiploid. In tumours in which the DNA analysis showed heterogeneity, the aneuploid DI value and corresponding SPF values were used in further analyses. The coefficient of variation (CV) was 4.4 ± 1.5% (mean ± s.d.). The CVs of Tru-cut biopsy specimens and surgical or transurethral biopsy specimens were 4.5 ± 1.6% and 4.3 ± 1.5% respectively.

### *Histological grading*

The histological grading of tumours was done according to the Gleason (1977) grading system. The samples were evaluated by one investigator who was unaware of the clinical data. The grading of 116 (116/325, 35%) tumours was based on Tru-cut biopsy specimens, and a mean of four Tru-cut biopsy specimens per tumour were available (range 2–6). The Tru-cut biopsy specimens contained mainly cancer tissue since Tru-cut biopsy specimens were taken from tumours that were already considered malignant on the basis of clinical examination. Accordingly, the grading of the tumours could be based on representative samples in all cases. The Gleason scores were 2–4 in 12 (18), 5–7 in 127 (149) and 8–10 in 134 (158) tumours. (The numbers in brackets indicate the number of cases in the original cohort.)

### *Statistical methods*

The basic statistical calculations were done using the SPSS-X program package on an IBM computer. The statistical tests used are indicated in the results when appropriate. Frequency distributions were tested by the chi-square test and Yates' correction was applied when necessary. The differences between the means of continuous variables were tested by analysis of variance. The univariate survival analysis (log-rank analysis, SPSS-X) was based on the life-table method with the statistics by Lee and Desu (1972). A multivariate survival analysis was done with the BMDP (2L) (Cox, 1972) in a stepwise manner and continuous variables were used as absolute numbers in this analysis. The enter limit was $P<0.1$, the remove limit was $P>0.15$ and deaths due to prostatic adenocarcinoma were used as events. The grouping of tumours according to their SPF was based on tertiles and on the median value. The multivariate analysis included only cases in which a complete set of data was available (FCM was not available in all cases). The year of treatment and patient age were included in the multivariate analysis to control their possible confounding effect on the results of biological variables. Univariate survival curves for T category and Gleason score are shown for the entire cohort (325 patients).

### Results

The FCM analysis showed that 158 of the 273 tumours (58%) were diploid and 115 (42%) aneuploid (non-diploid). Of the latter, 41 were tetraploid, three multiploid and 71 were non-tetraploid aneuploid. There were 297 diploid and 193 aneuploid tumour samples (in 54 tumours 3–5 samples were analysed). The SPF could not be calculated in four of the diploid and 12 of the aneuploid tumours. In the diploid tumours the SPF ranged from 0.2 to 18.9%, mean 4.0 ± 2.0 (s.d.), and in the aneuploid tumours from 1.6 to 31.9%, mean 11.8 ± 5.7 (s.d.). Thus, the mean SPF was nearly three times higher in the aneuploid tumours than in the diploid tumours. The mean (s.e.) SPF in Tru-cut biopsy specimens ($n = 81$) was 6.7% (0.6%), and in surgical or transurethral biopsy specimens ($n = 182$) 7.3% (0.4%) ($t$-test; $t = 0.87$, $P = 0.38$).

The relationship between T category, M category, Gleason score and DNA ploidy is shown in Table I. There were 165 T1–2M0–1 tumours, i.e. localised tumours with or without evidence of metastasis. Of these, 119 (72%) were diploid, whereas of the 108 T3–4M0–1, i.e. locally infiltrating tumours with or without metastases, only 39 (36%) were diploid. Eighty-four of the patients had distant metastases, and in only 26 (31%) of these patients were tumours diploid. The Gleason score was significantly related to DNA aneuploidy, as shown in Table I. Diploid tumours were more common (8/12, 67%) among tumours with a Gleason score

**Table I** The relationship between clinical stage, Gleason score and DNA ploidy

| *Variable* | *Number* | *Diploid (%)* | *Aneuploid (%)* | $\chi^2$, P |
|---|---|---|---|---|
| T1 | 101 | 84 | 16 | 50.3, $P<0.0001$ |
| T2 | 64 | 53 | 47 | |
| T3 | 82 | 36 | 64 | |
| T4 | 26 | 35 | 65 | |
| M0 | 189 | 70 | 30 | 36.1, $P<0.0001$ |
| M1 | 84 | 31 | 69 | |
| Gleason 2–4 | 12 | 67 | 33 | 41.4, $P<0.0001$ |
| Gleason 5–7 | 127 | 78 | 22 | |
| Gleason 8–10 | 134 | 39 | 61 | |

of 2–4, while aneuploidy was found in the majority of tumours (82/134, 61%) with Gleason scores of 8–10.

Intra-tumour heterogeneity of DI was found in only 2 of the 54 tumours (4%). There was also relatively little variation in SPF since the variation in 54 tumours from which multiple samples had been taken was 3.3 ± 3.4% (mean ± s.d.), which corresponds to a mean variation of 1.9% within each multiple sample.

SPF was significantly related to T and M categories so that T3–4 tumours and tumours with distant metastasis had high SPFs. Tumours with high Gleason scores also had a high SPF (Table II). DNA ploidy and SPF were independent of patient age ($P > 0.5$).

In univariate survival analysis, T category (Figure 1), M category ($\chi^2 = 107.6$, $P < 0.0001$), Gleason score (Figure 2), DNA ploidy (Figure 3) and SPF (Figure 4) were significant indicators of long-term prognosis. There was no significant difference ($\chi^2 = 0.2$, $P = 0.6$) in the 10 year survival between tetraploid (10 year survival 30%) and non-tetraploid aneuploid tumours (10 year survival 20%). Progression of T1–2M0 tumours during the follow-up was significantly related to Gleason score, DNA ploidy and SPF (Table III). In the survival analysis of T1–2M0 tumours, Gleason score ($\chi^2 = 13.4$, $P = 0.0013$), DNA ploidy (Figure 5) and SPF (Figure 6) were significantly related to prognosis. In patients with diploid T1M0 tumours ($n = 84$) the 10 year survival was 85%, in contrast to 65% in patients with aneuploid tumours ($n = 14$) ($\chi^2 = 4.8$, $P = 0.026$). The SPF showed a non-significant trend ($\chi^2 = 2.3$, $P = 0.12$).

**Table II** The S-phase fraction in various subgroups of prostatic adenocarcinoma

| *Variable* | *Number* | *SPF (s.e.)%* | *F/t*[a], P |
|---|---|---|---|
| T1 | 97 | 5.32 (0.42) | 6.9, $P < 0.0001$ |
| T2 | 63 | 6.26 (0.70) | |
| T3 | 79 | 9.18 (0.69) | |
| T4 | 25 | 9.14 (1.45) | |
| M0 | 184 | 6.25 (0.40) | − 3.83[a], $P = 0.0005$ |
| M1 | 80 | 9.13 (0.65) | |
| Gleason 2–4 | 11 | 7.49 (1.93) | 28.6, $P < 0.0001$ |
| Gleason 5–7 | 123 | 4.97 (0.36) | |
| Gleason 8–10 | 130 | 9.17 (0.55) | |
| Diploid | 156 | 3.94 (0.17) | 54.2, $P < 0.0001$ |
| Non-tetraploid aneuploid | 66 | 11.94 (0.81) | |
| Tetraploid | 38 | 11.63 (0.78) | |
| Multiploid | 3 | 12.30 (4.53) | |

[a]Three groups, analysis of variance; two groups, *t*-test.

**Figure 1** Survival of patients subdivided according to T category. The difference between the curves is significant ($\chi^2 = 92.1$, $P < 0.0001$). Curve A, T1, $n = 114$; curve B, T2, $n = 77$; curve C, T3, $n = 101$; curve D, T4, $n = 33$.

Tumours with a Gleason score of 5–7 could be separated into two prognostic groups on the basis of DNA ploidy ($\chi^2 = 4.0$, $P = 0.0439$) and median SPF (5%) ($\chi^2 = 11.5$, $P = 0.0007$). In patients with T1–2M0 tumours with Gleason score 5–7, the 10 year survival was 90% in those with diploid tumours ($n = 84$), and 45% in those with aneuploid tumours ($n = 18$, $\chi^2 = 14.9$, $P = 0.0001$). The survival of patients with an SPF below the median value of 5% ($n = 72$) was 90%, and of those with an SPF above the median ($n = 28$) was only 50% ($\chi^2 = 14.4$, $P = 0.0001$).

We defined a two-category system for prostatic adenocarcinoma in which Gleason score 2–4 and Gleason score 5–7 tumours with a diploid DNA histogram or SPF lower than 5% were combined into category A(1). Gleason score 8–10 tumours and the rest of Gleason score 5–7 tumours with aneuploid DI or an SPF above the median were combined into category A(2). The results of a survival analysis using this system (Table IV) indicate that this new classification is a highly significant prognostic factor.

The results of multivariate survival analysis in the entire cohort, in T1–4M0, in T1–2M0 and in T1M0 tumours are shown in Table V. If the Gleason score was not included in the analysis, the independent predictors were as shown in Table VI. If the new prognostic category (A) was included in the multivariate survival analyses, it included all the available

**Figure 2** Survival of patients subdivided according to Gleason score. The difference in survival is significant ($\chi^2 = 50.1$, $P < 0.0001$). Curve A, Gleason score 2–4, $n = 18$; curve B, Gleason score 5–7, $n = 149$; curve C, Gleason score 8–10, $n = 158$.

**Figure 3** Survival of patients subdivided according to DNA ploidy. The difference between the curves is significant ($\chi^2 = 31.0$, $P < 0.0001$). Curve A, diploid, $n = 158$; curve B, aneuploid, $n = 115$.

prognostic information in addition to T and M categories (Table VII).

In patients treated by oestrogens and orchiectomy independent predictors were M category ($P<0.001$), Gleason score ($P=0.002$), patient age ($P=0.063$) and T category ($P=0.066$), but if the Gleason score was not used in the analysis SPF was included among the independent predictors ($P=0.07$). In patients treated by transurethral resection M category ($P<0.001$), patient age ($P=0.021$) and T category ($P=0.038$) had independent prognostic value. In those patients subjected to active surveillance, T category ($P=0.002$) and DNA ploidy ($P=0.02$) were independent prognostic factors. If all the types of therapy were entered in multivariate analysis as binary data (yes/no), oestrogen therapy ($P=0.051$) and orchiectomy ($P=0.059$) resulted in

**Figure 4** Survival of the patients subdivided according to SPF. The difference in survival among the curves is significant ($\chi^2=35.3$, $P<0.0001$). Curve A, SPF <3.9%, $n=89$; curve B, SPF 3.9–6.6% $n=86$; curve C, SPF >6.6%, $n=89$.

**Figure 5** Survival of patients with a T1–2M0 tumour subdivided according to DNA ploidy. The difference in survival is significant ($\chi^2=9.5$, $P<0.002$). Curve A, diploid, $n=112$; curve B, aneuploid, $n=38$.

**Figure 6** Survival of patients with a T1–2M0 tumour subdivided according to median value of the SPF. The difference between the curves is significant ($\chi^2=12.1$, $P<0.0005$). Curve A, SPF <5%, $n=90$; curve B, SPF >5%, $n=57$.

**Table III** The progression of T1–2M0 tumours in relationship to Gleason score, DNA ploidy and SPF

| *Variable* | *Number* | *No. progression (%)* | *Progression (%)* | $\chi^2$, P |
|---|---|---|---|---|
| Gleason 2–4 | 14 | 86 | 14 | 9.42, $P=0.009$ |
| Gleason 5–7 | 114 | 81 | 19 | |
| Gleason 8–10 | 39 | 59 | 35 | |
| Diploid | 107 | 86 | 14 | 7.50, $P=0.006$ |
| Aneuploid | 34 | 65 | 35 | |
| SPF <3.9% | 62 | 87 | 13 | 9.7, $P=0.007$ |
| SPF 3.9–6.6% | 49 | 84 | 16 | |
| SPF >6.6% | 27 | 59 | 41 | |

**Table IV** Survival of prostatic adenocarcinoma subdivided according to category A1 and A2[a]

| | *Number* | *Alive at 5 years (%)* | *Alive at 10 years (%)* | $\chi^2$, P |
|---|---|---|---|---|
| *All cases* | | | | |
| Category A = 1 | 139 | 85 | 75 | |
| Category A = 2 | 186 | 50 | 25 | 41.5, $P<0.0001$ |
| *T3–4M0–1* | | | | |
| Category A = 1 | 21 | 45 | 20 | 0.1, $P=0.7$ |
| Category A = 2 | 113 | 35 | 15 | |
| *T1–4M0* | | | | |
| Category A = 1 | 119 | 90 | 85 | |
| Category A = 2 | 105 | 70 | 45 | 24.4, $P<0.0001$ |
| *T1–2M0* | | | | |
| Category A = 1 | 110 | 95 | 90 | |
| Category A = 2 | 64 | 75 | 50 | 21.9, $P<0.0001$ |

[a]Category A is 1 if Gleason score is 2–4 or score 5–7 tumour is diploid. Category A is 2 if Gleason score is 8–10 or score 5–7 tumour is aneuploid. The results are similar if SPF 5% is used as a cut-off limit.

**Table V** Results of the multivariate survival analysis with Gleason score included

| | *β(s.e.)* | P-*value* | *Hazard rate (95% CI)* |
|---|---|---|---|
| *All cases* | | | |
| M category | 1.096 (0.240) | <0.001 | 2.99 (1.85–4.83) |
| Gleason score | 0.710 (0.229) | <0.001 | 2.03 (1.29–3.21) |
| T category | 0.478 (0.134) | 0.003 | 1.61 (1.23–2.11) |
| Age | 0.042 (0.012) | 0.001 | 1.04 (1.02–1.07) |
| SPF | 0.024 (0.013) | 0.087 | 1.02 (1.00–1.05) |
| *T1–4M0* | | | |
| T category | 0.777 (0.174) | <0.001 | 2.17 (1.53–3.08) |
| Age | 0.057 (0.018) | 0.001 | 1.06 (1.02–1.10) |
| Gleason score | 0.925 (0.331) | 0.004 | 2.52 (1.30–4.89) |
| *T1–2M0* | | | |
| Gleason score | 0.791 (0.396) | <0.001 | 2.20 (1.00–4.87) |
| T category | 0.969 (0.405) | 0.022 | 2.64 (1.17–5.92) |
| SPF | 0.069 (0.033) | 0.058 | 1.07 (1.00–1.14) |
| *T1M0* | | | |
| Gleason score | 1.613 (0.587) | 0.008 | 5.02 (1.55–16.23) |

Hazard rate = $e^{\beta}$; CI, confidence interval.

**Table VI** Results of the multivariate survival analysis with Gleason score excluded

| | β (s.e.) | P-value | Hazard rate (95% CI) |
|---|---|---|---|
| *All cases* | | | |
| M category | 1.096 (0.238) | <0.001 | 2.99 (1.85-4.81) |
| T category | 0.646 (0.124) | <0.001 | 1.90 (1.48-2.44) |
| Age | 0.042 (0.013) | 0.002 | 1.04 (1.01-1.07) |
| SPF | 0.030 (0.013) | 0.031 | 1.03 (1.00-1.06) |
| *T1-4M0* | | | |
| T category | 0.969 (0.159) | <0.001 | 2.63 (1.91-3.62) |
| Age | 0.056 (0.019) | 0.001 | 1.05 (1.02-1.09) |
| DNA ploidy | 0.529 (0.306) | 0.086 | 1.69 (0.92-3.12) |
| *T1-2M0* | | | |
| T category | 1.253 (0.384) | 0.001 | 3.50 (1.62-7.54) |
| SPF | 0.086 (0.028) | 0.006 | 1.09 (1.03-1.15) |
| *T1M0* | | | |
| DNA ploidy | 1.730 (0.643) | 0.015 | 5.64 (1.58-20.0) |

Hazard rate = $e^{\beta}$; CI, confidence interval.

**Table VII** Results of the multivariate survival analysis when the novel prognostic category (A = 1 or 2)[a] is included

| | β (s.e.) | P-value | Hazard rate (95% CI) |
|---|---|---|---|
| *All cases* | | | |
| M category | 1.222 (0.238) | <0.001 | 3.39 (2.11-5.46) |
| Category A | 1.104 (0.295) | <0.001 | 3.02 (1.67-5.44) |
| T category | 0.361 (0.133) | 0.008 | 1.43 (1.10-1.87) |
| *T1-4M0* | | | |
| Category A | 1.569 (0.404) | <0.001 | 4.80 (2.14-10.77) |
| T category | 0.515 (0.156) | 0.001 | 1.67 (1.22-2.29) |
| *T1-2M0* | | | |
| Category A | 2.167 (0.462) | <0.001 | 8.73 (3.46-21.99) |
| *T1M0* | | | |
| Category A | 2.504 (0.680) | <0.001 | 12.23 (3.27-45.78) |

[a]See the footnote in Table IV.

lowered survival probability, while other types of therapy had no independent prognostic significance.

### Discussion

Heterogeneity of DNA indices is a common feature of malignant neoplasms. The fraction of tumours showing heterogeneous DNA indices in FCM varies usually between 20% and 40% (Kallioniemi, 1988; Lipponen *et al.*, 1993), although up to 90% of lung tumours may show heterogeneous DNA indices (Carey *et al.*, 1990). A high proportion of prostatic adenocarcinomas have been reported to have heterogeneous DNA indices in image cytometry (Nagel & Al-Abadi, 1991). In the present FCM analysis only 4% of the tumours showed intra-tumour variability in DI, and the mean variation of SPF within a tumour was also low. Accordingly, the results suggest that intra-tumour heterogeneity of DNA ploidy and SPF is a rare phenomenon in prostatic adenocarcinoma, in contrast to several other epithelial neoplasms (Kallioniemi, 1988; Carey *et al.*, 1990; Fernö *et al.*, 1992; Lipponen *et al.*, 1993). When interpreting the results of heterogeneity analysis one should note that the sampling of tissue was not completely random, which may skew the results. However, the results give a good estimate of the overall reproducibility of the DNA measurements in this cohort of patients. Comparative studies between different laboratories have shown that ploidy classifications may vary in 10-15% of tumours when the measurements are taken from adjacent tissue sections in prostatic carcinoma (Fosså *et al.*, 1992). However, it cannot be excluded that part of this variation has a biological basis since tumours are often highly heterogenous in their cellular features.

DNA aneuploidy was related to invasive high-grade disease, which is in agreement with previous reports in prostatic adenocarcinoma (Jones *et al.*, 1990; Eskelinen *et al.*, 1991; Tribukait, 1991; Visakorpi *et al.*, 1991; Di Silvero *et al.*, 1992). The present results confirm previous observations in that tetraploidy is related to intermediate or high stages (Eskelinen *et al.*, 1991; Tribukait, 1991), while the fraction of tumours with multiple cell lines was clearly lower than in some previous reports (Tribukait, 1991). However, there was no difference in the prognostic value between various levels of aneuploidy, as reported by some investigators (Hedlund *et al.*, 1988; Tribukait, 1991). The present results are in accord with the results in other epithelial carcinomas (Frierson, 1991; Lipponen *et al.*, 1993), in which the degree of aneuploidy as measured by flow cytometry had no additional prognostic value.

The SPF values were similar in Tru-cut biopsy specimens and in surgical or transurethral resection specimens, and accordingly the type of specimen was not a significant confounding factor, although Fåssa *et al.* (1992) reported some differences in FCM results in different types of biopsy. Aneuploid tumours showed significantly higher SPF values than the diploid tumours, which is in accord with previous results (Eskelinen *et al.*, 1991; Visakorpi *et al.*, 1991). The degree of aneuploidy had little influence on SPF value, which further supports the limited prognostic value of the degree of aneuploidy. In addition, the clinical correlations in this analysis and in previous reports (Eskelinen *et al.*, 1991; Visakorpi *et al.*, 1991) suggest that the SPF is a more important prognostic indicator than ploidy. In other epithelial tumours, the proliferation rate of cancer cells has been included, in addition to clinical stage, among the independent predictors (Frierson, 1991; Lipponen *et al.*, 1993). The proliferation of cells is regulated through specific genes (Visakorpi *et al.*, 1992), and aneuploidy may be a reflection of genetic instability rather than proliferative potential.

Histopathological grading has been shown to correlate with stage and prognosis in prostatic adenocarcinoma (Mostofi, 1975; Gleason, 1977; Oesterling *et al.*, 1987; Badalament *et al.*, 1991; Visakorpi *et al.*, 1991). However, tumours with intermediate differentiation are biologically heterogeneous (Nagel & Al-Abadi, 1991), and accordingly the prognosis of patients with these tumours is also variable. In this analysis, tumours with Gleason scores 5-7 could be regrouped into distinctly different prognostic groups by FCM, which suggests a clinical application for FCM in prostatic adenocarcinoma. The importance of this classification could be confirmed in multivariate analysis, which clearly showed that neither the original Gleason score nor FCM data were included among the independent predictors. Tumours with Gleason scores of 2-4 or 8-10 could not be subdivided into prognostic groups by determining DI or SPF, since nearly all the low-score tumours are diploid and the high-score tumours aneuploid. This association is reflected also in the results of analysis of T3-4 tumours, which could not be regrouped. The combined use of quantitative measurements and subjective histological assessment has been previously tested in transitional cell bladder tumours with similar results (Lipponen *et al.*, 1992).

Although patient age and malignant histological features (Gleason score, Di, SPF, clinical stage) were not significantly interrelated, patient age was an independent prognostic factor in all T3-4 tumours. This suggests that compromised general physical condition and reduced capacity to resist tumour growth are of importance, as has been observed in other neoplastic diseases (Aaltomaa *et al.*, 1991). Most of the previous survival analyses performed in prostatic carcinoma have not considered age as a prognostic factor (Stephenson *et al.*, 1987; Badalament *et al.*, 1991; Eskelinen *et al.*, 1991; Nagel & Al-Abadi, 1991; Tribukait, 1991; Di Silvero *et al.*, 1992; Peter-Gee *et al.*, 1992), and accordingly the comparison of results based on biological variables alone is difficult. The year of treatment was included in the multivariate analysis but did not relate to prognosis.

To test the possibility that the type of therapy might influence the prognosis, therapy was included in separate multivariate analyses, which showed that none of the therapies improved the prognosis in relation to clinical, histological and FCM parameters. In contrast, hormonal therapy was related to an unfavourable prognosis, which is probably because of the preferential use of hormones in patients with disseminated disease. Separate analyses of three major therapy groups (hormones, electroresection, active surveillance) showed that the same parameters had independent prognostic significance as in the entire cohort. Accordingly, the results of these analyses suggest that the biological characteristics of prostatic tumours are most important for the outcome of the patients.

In summary, we conclude that DNA aneuploidy and a high SPF are related to a malignant phenotype in prostatic adenocarcinomas. In practice, FCM can be used to subdivide tumours with Gleason scores of 5-7 into subgroups with different prognoses. The combination of FCM, conventional histological grading and clinical or histological staging provides accurate prognostic information in prostatic adenocarcinomas.

This study was supported by a grant from Savo Cancer Fund. The technical assistance of Ms Monica Schoultz is gratefully acknowledged.

**References**

AALTOMAA, S., LIPPONEN, P., ESKELINEN, M., KOSMA, V.-M., MARIN, S., ALHAVA, E. & SYRJÄNEN, K. (1991). Prognostic scores combining clinical, histological and morphometric variables in assessment of the disease outcome in female breast cancer. *Int. J. Cancer*, **49**, 886-892.

ADOLFSSON, J. & TRIBUKAIT, B. (1991). Modal DNA-values in prostate cancer patients with deferred therapy or endocrine therapy. *Acta Oncol.*, **30**, 209-214.

BADALAMENT, R.A., O'TOOLE, R.V., YOUNG, D.C. & DRAGO, J.R. (1991). DNA ploidy and prostate-specific antigen as prognostic factors in clinically resectable prostate cancer. *Cancer*, **67**, 3014-3023.

BAISCH, H., GÖHDE, W. & LINDEN, W.A. (1975). Analysis of PCP data to determine the fraction of cells in various phases of cell cycle. *Radiat. Environ. Biophys.*, **12**, 31-39.

CAMPLEJOHN, R.S., MACARTNEY, J.C. & MORRIS, R.W. (1989). Measurement of S-phase fractions in lymphoid tissue comparing fresh versus paraffin-embedded tissue and 4'-6'-diamino-2 phenylindole dihydrochloride versus propidium iodide staining. *Cytometry*, **10**, 410-416.

CAREY, F.A., LAMB, B.A.O. & BIRD, C.C. (1990). Intratumoral hetergeneity of DNA content in lung cancer. *Cancer*, **65**, 2266-2269.

COX, D.R. (1972). Regression models and life tables with discussion. *J. R. Stat. Soc. B.*, **34**, 187-220.

DEITCH, A.D. & DEVERE WHITE, R.W. (1992). Flow cytometry as a predictive modality in prostate cancer. *Hum. Pathol.*, **23**, 352-359.

DI SILVERO, F., D'ERAMO, G., CAPONERA, M., PERSECHINO, F., ELEUTERI CAVALLO, D., DE VITA, R. & FORTE, D. (1992). The prognostic value of DNA content in patients with prostatic carcinoma. *Eur. Urol.*, **21**, 92-95.

ESKELINEN, M., LIPPONEN, P., MAJAPURO, R., SYRJÄNEN, K. & NORDLING, S. (1991). DNA ploidy, S-phase fraction and G2 fraction as prognostic determinants in prostatic adenocarcinoma. *Eur. Urol.*, **19**, 274-278.

FOSSÅ, S.D., BERNER, A., WAEHRE, H., HEIDEN, T., HOLM JUUL, M.E., VAN DEN OUDEN, D., PETTERSEN, E.O., WANG, N. & TRIBUKAIT, B. (1992). DNA ploidy in cell nuclei from paraffin-embedded material - comparison of results from two laboratories. *Cytometry*, **13**, 395-403.

FERNÖ, M., BALDETORP, B., EWERS, S.-B., IDVALL, I., OLSSON, H., SIGURDSSON, H. & KILLANDER, D. (1992). One or multiple samplings for flow cytometric DNA analyses in breast cancer - prognostic implications? *Cytometry*, **13**, 241-249.

FRIERSON, Jr, H. (1991). Ploidy analysis and S phase fraction determination by flow cytometry of invasive carcinoma of the breast. *Am. J. Surg. Pathol.*, **15**, 358-367.

GAETA, J.F., ASIRWATHAM, J.E., MILLER, G. & MURPHY, G.P. (1980). Histologic grading of primary prostatic cancer: a new approach to an old problem. *J. Urol.*, **123**, 689-693.

GLEASON, D.F. (1977). Histologic grading and clinical staging of prostatic carcinoma. In *Urologic Pathology: The Prostate*. Tannenbaum, M. (ed.) p. 171. Lea & Febiger: Philadelphia.

HEDLUND, P.O., ADOLFFSSON, J., RÖNSTRÖM, L. & TRIBUKAIT, B. (1988). Modal DNA as prognostic indicator in untreated prostatic carcinoma. *Scand. J. Urol. Nephrol.*, **110**, 125-129.

JONES, E.C., MCNEAL, J., BRUCHOVSKY, N. & DE JONG, G. (1990). DNA content in prostatic adenocarcinoma. A flow cytometry study of the predictive value of aneuploidy for tumour volume, percentage Gleason grade 4 and 5, and lymph node metastases. *Cancer*, **66**, 752-757.

KALLIONIEMI, O.-P. (1988). Comparison of fresh and paraffin-embedded tissue as starting material for DNA flow cytometry and evaluation of intratumour heterogeneity. *Cytometry*, **9**, 164-169.

LEE, E. & DESU, M. (1972). A computer program for comparing k samples with right censored data. *Computer Programs Biomed.*, **2**, 315-318.

LIPPONEN, P.K., ESKELINEN, M.J., JAUHIAINEN, K., HARJU, E., TERHO, R. & HAAPASALO, H. (1992). Prognostic factors in WHO grade 2 transitional cell bladder cancer (TCC): a novel two grade classification system for TCC based on mitotic index. *J. Cancer Res. Clin. Oncol.*, **118**, 615-620.

LIPPONEN, P., NORDLING, S., ESKELINEN, M.J., JAUHIAINEN, K., TERHO, R. & HARJU, E. (1993). Flow cytometry in comparison with mitotic index in predicting disease outcome in transitional cell bladder cancer. *Int. J. Cancer*, **53**, 42-47.

MOSTOFI, F.K. (1975). Grading of prostatic carcinoma. *Cancer Chemother. Rep.*, **59**, 111-117.

MUIR, C.S., NECTOUX, J. & STASZEWSKI, J. (1991). The epidemiology of prostatic cancer. *Acta Oncol.*, **30**, 133-140.

NAGEL, R. & AL-ABADI, H. (1991). The prognostic significance of ploidy and DNA heterogeneity in the primary diagnosis and monitoring of patients with locally advanced prostatic carcinoma. *Scand. J. Urol. Nephrol.*, **138**, 83-92.

OESTERLING, J.E., BRENDLER, C.B., EPSTEIN, J.I., KIMBALL, A.W. & WALSH, P.C. (1987). Correlation of clinical stage, serum prostatic acid phosphatase and preoperative gleason grade with final pathological stage in 275 patients with clinically localized adenocarcinoma of the prostate. *J. Urol.*, **138**, 92-98.

PETERS-GEE, J.M., MILES, B.J., CERNY, J.C., GABA, A.R., JACOBSEN, G. & CRISSMAN, J.D. (1992). Prognostic significance of DNA quantitation in stage D1 prostate carcinoma with the use of image analysis. *Cancer*, **70**, 1159-1165.

ROBERTSON, C.N. & PAULSON, D.F. (1991). DNA in radical prostatectomy specimens: prognostic value of tumour ploidy. *Acta Oncol.*, **30**, 205-207.

SHANKEY, T.V., KALLIONIEMI, O.-P., KOSLOWSKI, J.M., LIEBER, M.L., MAYHALL, B.H., MILLER, G. & SMITH, G.J. (1993). Consensus review of the clinical utility of DNA content cytometry in prostatic cancer. *Cytometry*, **14**, 497-500.

SONG, J., CHENG, W.S., CUPPS, R.E., EARLE, J.D., FARROW, G.M. & LIEBER, M.M. (1992). Nuclear deoxyribonucleic acid content measured by static cytometry: important prognostic association for patients with clinically localized prostate carcinoma treated by external beam radiotherapy. *J. Urol.*, **147**, 794-797.

STEPHENSON, R.A., JAMES, B.C., GAY, H., FAIR, W.R., WHITMORE, Jr, W.F. & MELAMED, M.R. (1987). Flow cytometry of prostate cancer: relationship of DNA content to survival. *Cancer Res.*, **47**, 2504-2509.

TRIBUKAIT, B. (1991). DNA flow cytometry in carcinoma of the prostate for diagnosis, prognosis and study of tumour biology. *Acta Oncol.*, **30**, 187-192.

UICC (1978). *TNM Classification of Malignant Tumours*, 3rd edn. UICC: Geneva.

VISAKORPI, T., KALLIONIEMI, O.-P., PARONEN, I.Y.I., ISOLA, J.J., HEIKKINEN, A.I. & KOIVULA, T.A. (1991). Flow cytometric analysis of DNA ploidy and S phase fraction from prostatic carcinomas: implications for prognosis and response to endocrine therapy. *Br. J. Cancer*, **64**, 578-582.

VISAKORPI, T., KALLIONIEMI, O.-P., HEIKKINEN, A., KOIVULA, T. & ISOLA, J. (1992). Small subgroup of aggressive highly proliferative prostatic carcinomas defined by p53 accumulation. *J. Natl Cancer Inst.*, **84**, 883-887.

WIRTH, M.P., MULLER, H.A., MANSECK, A., MULLER, J. & FROHMULLER, H.G.W. (1991). Value of nuclear DNA ploidy patterns in patients with prostate cancer after radical prostatectomy. *Eur. Urol.*, **20**, 248-252.

ZETTERBERG, A. & FORSSLUND, G. (1991). Ploidy level and tumour progression in prostatic carcinoma. *Acta Oncol.*, **30**, 193-199.